# A simple and rapid method to characterize lipid fate in skeletal muscle

**DOI:** 10.1186/1756-0500-7-391

**Published:** 2014-06-24

**Authors:** Julie Massart, Juleen R Zierath, Alexander V Chibalin

**Affiliations:** 1 Department of Molecular Medicine and Surgery, Section for Integrative Physiology, Karolinska Institutet, Stockholm, Sweden; 2 Department of Physiology and Pharmacology, Section for Integrative Physiology, Karolinska Institutet, Stockholm, Sweden

**Keywords:** Skeletal muscle, Free fatty acids, Lipid metabolism, Thin-layer chromatography

## Abstract

**Background:**

Elevated fatty acids contribute to the development of type 2 diabetes and affect skeletal muscle insulin sensitivity. Since elevated intramuscular lipids and insulin resistance is strongly correlated, aberrant lipid storage or lipid intermediates may be involved in diabetes pathogenesis. The aim of this study was to develop a method to determine the dynamic metabolic fate of lipids in primary human skeletal muscle cells and in intact mouse skeletal muscle. We report a simple and fast method to characterize lipid profiles in skeletal muscle using thin layer chromatography.

**Findings:**

The described method was specifically developed to assess lipid utilization in cultured and intact skeletal muscle. We determined the effect of a pan-diacylglycerol kinase (DGK) class I inhibitor (R59949) on lipid metabolism to validate the method. In human skeletal muscle cells, DGK inhibition impaired diacylglycerol (DAG) conversion to phosphatidic acid and increased triglyceride synthesis. In intact glycolytic mouse skeletal muscle, DGK inhibition triggered the accumulation of DAG species. Conversely, the DGK inhibitor did not affect DAG content in oxidative muscle.

**Conclusion:**

This simple assay detects rapid changes in the lipid species composition of skeletal muscle with high sensitivity and specificity. Determination of lipid metabolism in skeletal muscle may further elucidate the mechanisms contributing to the pathogenesis of insulin resistance in type 2 diabetes or obesity.

## Findings

### Background

Dysregulation of lipid metabolism, leading to lipid content modification as well as production of second messengers, contribute to the pathogenesis of insulin resistance in type 2 diabetes and obesity. Following uptake in skeletal muscle, free fatty acids (FFA) are converted to long-chain fatty acyl-CoAs (LCACoAs), which can undergo several fates. LCACoAs can be imported into the mitochondria and used as substrates for β-oxidation, incorporated into triglycerides, or serve as a source of second messengers, such as diacylglycerol (DAG). In skeletal muscle from obese humans [1,2], FFA oxidation capacity is reduced, thereby leading to intramuscular triacylglycerol accumulation [3,4]. In addition, an accumulation of secondary messenger lipid species such as DAG or ceramide, also contributes to muscle insulin resistance, thereby exacerbating the severity of type 2 diabetes [5-7]. For example, DAG accumulation activates specific protein kinase C isoform activity and impairs insulin-stimulated glucose transport through enhanced IRS-1 serine phosphorylation [8]. Understanding how FFA levels impact glucose metabolism may elucidate the role of lipids in the pathogenesis insulin resistance in type 2 diabetes or obesity. Here, we present a simple and fast method to characterize the metabolic fate of lipids in skeletal muscle using a thin layer chromatography system (TLC).

### Primary human skeletal muscle cell culture

Satellite cells were isolated from *vastus lateralis* skeletal muscle biopsies derived from healthy volunteers by trypsin-EDTA digestion, as previously described [9]. All participants provided written informal consent and all protocols were approved by the Karolinska Institutet ethics committee. Myoblasts were propagated in growth medium (F12/DMEM, 20% FBS, 1% PeSt and 1% fungizone (Invitrogen, Sweden)), and differentiated at >80% confluence in low-serum medium (DMEM containing 1 g/l glucose, 2% FBS, 1% PeSt and 1% Fungizone). Experiments were performed on differentiated myotubes cultured in 6-well plates. Final experiments were conducted 7 days after differentiation was induced.

Cultured primary human skeletal muscle cells were incubated with 0.2 μCi/ml [^14^C(U)] palmitate (Perkin Elmer, CA, USA) with non-radioactive palmitate (25 nM) for 6 hours in the presence or absence of the DAG kinase inhibitor (R59949, Calbiochem, Merck AB, Sweden). Following the incubation step, cells were washed 3 times with cold PBS in order to remove the free and membrane-bound radioactive palmitate.

### Mouse muscle incubation

Male C57BL/6 mice were purchased from Charles River (Germany). Mice were housed on a 12 hour light/dark cycle and received *ad libitum* standard rodent chow. Experiments were approved by the Regional Animal Ethical Committee (Stockholm, Sweden).

Mice (12–14 weeks old) were fasted for 4 hours prior to the study. Mice were anesthetized intraperitoneally with Avertin (2,2,2-tribromoethanol and tertiary amyl alcohol) at a volume of 10 μl/g body weight. Extensor digitorum longus (EDL) and soleus muscles were carefully dissected without stretching and gently removed with tendons intact. Muscles were incubated for 30 minutes at 30°C in vials containing pre-oxygenated (95% O_2_, 5% CO_2_) Krebs-Henseleit buffer (KHB) supplemented with 15 mM mannitol, 5 mM glucose, 3.5% fatty acid-free bovine serum albumin and 0.3 mM palmitate. Muscles were then transferred to new vials containing fresh pre-gassed KHB, supplemented as described above containing 2.5 μCi/ml of [^14^C(U)]-palmitate, and incubated for 120 min in the presence or absence of 25 μM DAG kinase inhibitor (R59949). At the end of the incubation, tendons were removed from muscles, which were rapidly weighed, immediately frozen in liquid nitrogen, and stored at -80°C.

### Lipid extraction

Cultured cells were scraped directly from plates in 300 μl of an isopropanol/ 0.1% acetic acid mixture. Frozen muscles were disrupted in the same buffer using the TissueLyser II (Qiagen). The samples were incubated overnight at room temperature with slight shaking to allow lipids to diffuse into the solvent. Next, 600 μl of hexane and 150 μl of 1 M KCl were added to each sample. The hexane-isopropanol system is particularly suitable for extraction of hydrophobic lipids, such as free fatty acids, triglycerides and cholesterol esters [10]. Addition of KCl is designed to improve the removal of non-lipid contaminants [11], including proteins and amino acids. Samples were then rotated for 10 minutes at room temperature. Tubes were stored upright for 5 minutes to induce phase separation. The organic phase (upper phase) was collected (~600 μl) and transferred to a new tube. The organic phase was dried using a vacuum pump for 1 hour. Alternatively, nitrogen steam could be used to dry the lipids. The lipid pellet was eluted in 50 μl of 1:1 methanol:chloroform.

### Detection of lipid species with thin layer chromatography

TLC plates that contain a concentration zone and are channeled were selected to facilitate the loading of lipid extracts (Silica Gel G 250 μm 20×20 cm, Analtech, DE, USA). One hour before the development of the TLC plate, the loading chamber was filled with 100 ml of a hexane:diethylether:acetic acid mixture (80:20:3). The lid of the chamber was then sealed using high-vacuum grease (Corning, NY, USA) to allow vapor to accumulate in the chamber. The lipid suspension was then applied to the TLC plate at ~2 cm from the bottom (on the preadsorbant zone) and separated in the hexane:diethylether:acetic acid system for 30 minutes. The compounds 1,2-Dioctanyl [1-^14^C] rac-glycerol (1,2-DAG), 1,3-Dioleoyl-rac-glycerol [oleoyl-1-^14^C] (1,3-DAG) (American Radiolabeled Chemicals Inc, MO, USA), [U-^14^C]palmitate (Perkin Elmer), and [^14^C]triolein (TG) were used as standards. Plates were dried for 5–10 minutes (until all solvents evaporated) and wrapped in plastic foil. The wrapped plate was transferred to an exposure cassette (GE Healthcare) and exposed to an X-ray film. The cassette was also wrapped with plastic in order to avoid moisture damage and was stored at -80˚C overnight.

### Quantification

After overnight incubation, the cassette was removed from -80˚C and allowed to stand at room temperature for 1 hour. The X-ray film was developed using an X-ray developer machine. Quantification by densitometry was performed using Quantity One software (Biorad).

### Detection of fatty acid oxidation with ^3^H-palmitate

In addition to lipid intermediates, the described protocol can also be used to simultaneously detect fatty acid oxidation, through the addition of ^3^H-palmitate along with ^14^C-palmitate at the same time. For this, the TLC plate should be sprayed with an autoradiographic enhancer spray (EN^3^HANCE, Perkin Elmer) prior to X-ray film exposure. Application of EN^3^HANCE should be repeated 3 times for 10 seconds, with complete drying of the TLC plate following each round. This spray allows the detection of lowly abundant species and can reduce the time necessary for sufficient exposure. Fatty acid oxidation is measured by the release of ^3^H_2_O in the media [12].

## Results and discussion

**Figure 1:**
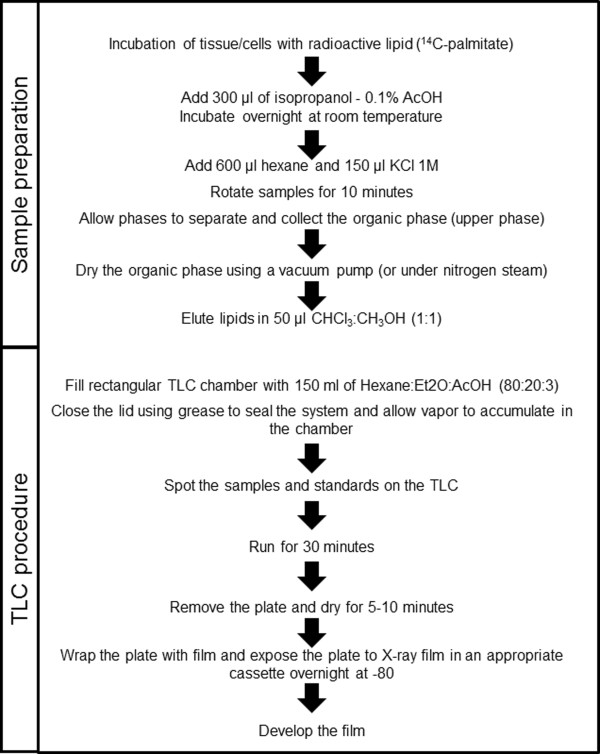
**Schematic representation of lipid extraction and TLC development.** The outlined protocol was established to extract, separate and identify lipid species in primary human skeletal muscle cells and intact murine skeletal muscles.

**Figure 2:**
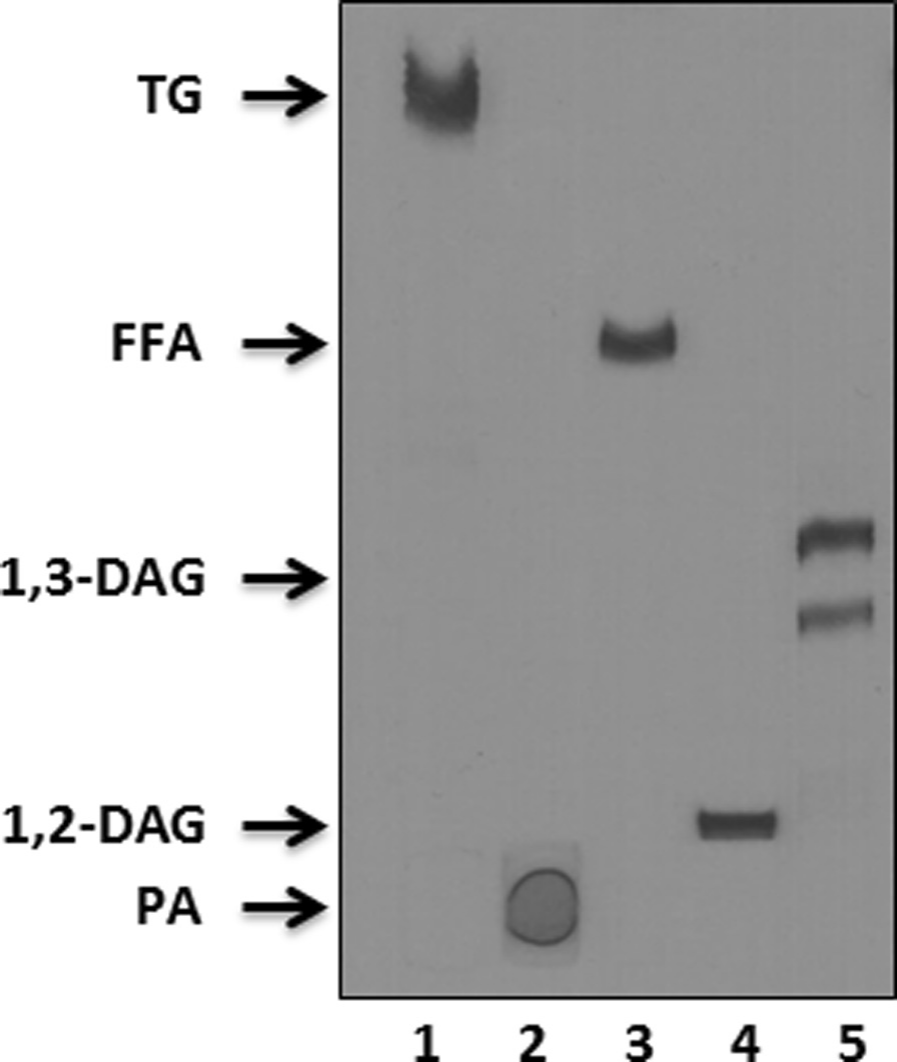
**Representative image of the separation of the standards.** Triglycerides (Lane 1), phosphatidic acid (Lane 2), free fatty acids (Lane 3), 1,2-DAG (Lane 4) and 1,3-DAG (Lane 5).

A schematic representation of the protocol used to identify lipid species in skeletal muscle by TLC is described in Figure 1. Separation of lipid standard samples from the origin is shown in Figure 2. Phosphatidic acid cannot enter the plate and appears at the origin, while 1,2-DAG migrated a few millimeters above the origin. The two distinct bands of 1,3-DAG are visible at 2 and 3 cm above the origin, due to the conformation of the lateral chains. Palmitic acid and triglycerides migrated at 6 cm and 9 cm, respectively, from the origin. The characterization of the lipid standard profile is crucial, since standards can be pooled together in one lane to allow more samples to be run on the same plate.

**Figure 3:**
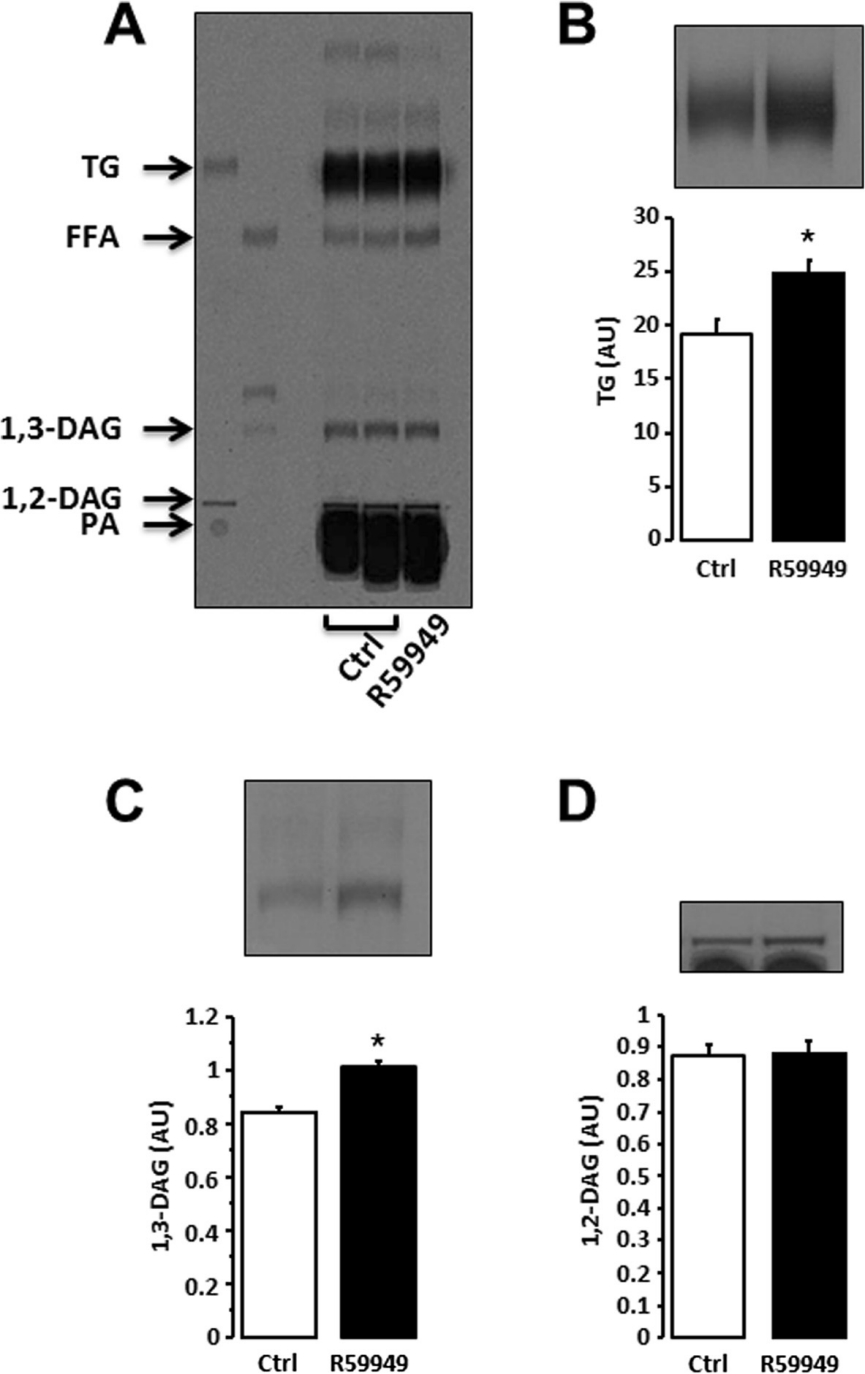
**Lipid profile in cultured human skeletal muscle cells.** Representative image of the lipid profile in cultured human skeletal muscle cells with lipid standards: FFA (palmitate), phosphatidic acid (PA), diacylglycerol (1,2- and 1,3-DAG) and triglycerides (TG) **(A)**. Quantification of triglycerides **(B)**, 1,2-DAG **(C)**, 1,3-DAG **(D)** in absence (Ctrl: control) or presence of DGK inhibitor (R59949). n = 3 independent experiments performed in duplicate. *p < 0.05 versus control. Results are means ± SEM.

**Figure 4:**
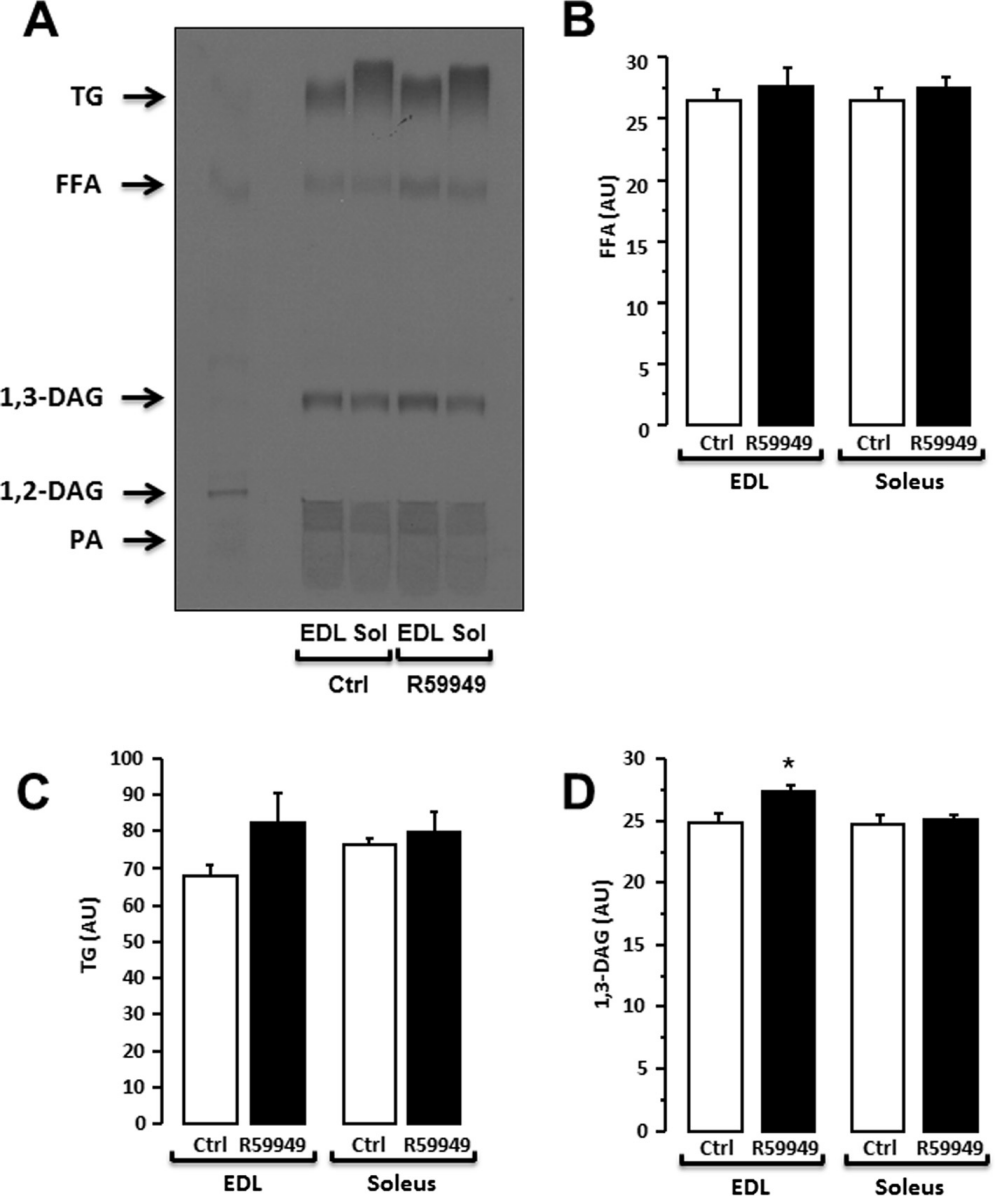
**Lipid profile in intact mouse skeletal muscle.** Representative image of the lipid profile in mouse EDL and soleus muscle in absence or in presence of a diacylglycerol kinase inhibitor (Ctrl: Control ; R59949: DGK inhibitor) **(A)**. Free fatty acids (FFA), phosphatidic acid (PA), diacylglycerol (1,2- and 1,3-DAG) and triglycerides (TG) are used as standards. Quantification of free fatty acids **(B)**, triglycerides **(C)** and 1,3-DAG **(D)** in absence (Ctrl: control) or presence of DGK inhibitor (R59949). n = 5. *p < 0.05 versus control. Results are means ± SEM.

Exposure of primary human muscle cells to ^14^C-palmitate for 6 hours produced a lipid migration pattern as shown in Figure 3A. To further validate the sensitivity and specificity of this system, primary human muscle cells were treated with a specific DAG kinases inhibitor (R59949; Figure 3B-D). DGK inhibition increased by 31% 1,3-DAG (Figure 3C) and by 30% percent triglycerides species compared with the control (Figure 3B) in primary human myotubes, which reflects an impaired DAG conversion to phosphatidic acid. This result is consistent with previous published work [13], confirming the sensitivity of the assay. Different exposure times can be easily selected to quantify different lipid species according to their abundance.The metabolic fate of free fatty acids was also determined in intact mouse skeletal muscle (Figure 4A). Treatment of mouse glycolytic EDL skeletal muscle with a DGK inhibitor led to an accumulation of 1,3-DAG species (Figure 4D). However the DGK inhibitor did not alter 1,3-DAG species in oxidative soleus muscle. In contrast to human cells, triglyceride synthesis in mouse EDL muscle remained unchanged after incubation with the DGK inhibitor (Figure 4C), possibly due to a reduced incubation time.

## Conclusion

Different systems exist to explore cellular lipid profiles in various biological systems, including high-performance liquid chromatography, gas chromatography, and mass spectrometry. TLC is a convenient system that allows simple and easy determination and quantification of lipid species. The equipment required for TLC is quite inexpensive and the experimental procedure can be quickly established in most laboratory environments.

The advantages of TLC and different applications have been reviewed earlier [14,15]. Lipid extraction using isopropanol/hexane was first used by Hara and Radin [11] as an alternative to chloroform/methanol extraction and extracts a high percentage of the lipids with low protein contamination. Moreover, use of plastic materials is possible with this extraction procedure. The possibility to use alternative TLC buffer systems allows the user to specifically separate the lipid species of interest [16,17]. Finally, the use of a single mobile phase permits simultaneous comparisons of multiple samples. Hence, this method provides an easy and rapid evaluation of how exogenous compounds influence lipid metabolism.

In developing the current TLC protocol, radioactive palmitate was used as a convenient, inexpensive, and available physiological substrate. Due to the great diversity in fatty acid structure, with varying chain length or degree of saturation, different substrates (e.g. oleate, myristate, laurate) can assume distinct fates. Therefore, different radiolabeled fatty acids can be used in the same TLC system in order to examine the effects of exogenous compounds on lipid abundance. In conclusion, this method allows for the easy, fast and efficient detection of changes in lipid metabolism in both cultured and intact skeletal muscle. Moreover, this method can readily be extrapolated to other cell types and tissues such as brain, heart, liver or smooth muscle.

## Abbreviations

AcOH: Acetic acid; DAG: Diacylglycerol; DGK: Diacylglycerol kinase; EDL: Extensor digitorum longus; KHB: Krebs-Henseleit buffer; TLC: Thin layer chromatography; TG: Triglycerides.

## Competing interests

The authors declare no competing interests.

## Authors’ contributions

JM designed and performed the cell culture and animal experiments. JM and AVC interpreted the data. JM, AVC and JRZ wrote the manuscript. All authors read and approved the final manuscript.

## Authors’ information

Our research group focuses on cellular mechanisms underlying the development of insulin resistance in Type 2 diabetes, as well as delineation of exercise-mediated effects on skeletal muscle glucose metabolism and gene expression.
